# Supplementary material for: NHSL3 controls single and collective cell migration through two distinct mechanisms
Source: Nat Commun. 2025 Jan 2;16:205. doi: 10.1038/s41467-024-55647-3 (PMC11696792; doi:10.1038/s41467-024-55647-3)

Supplementary Information

SUPPLEMENTARY FIGURES 2
ANNEX : UNCROPPED WESTERN BLOTS 11

Supplementary Figures

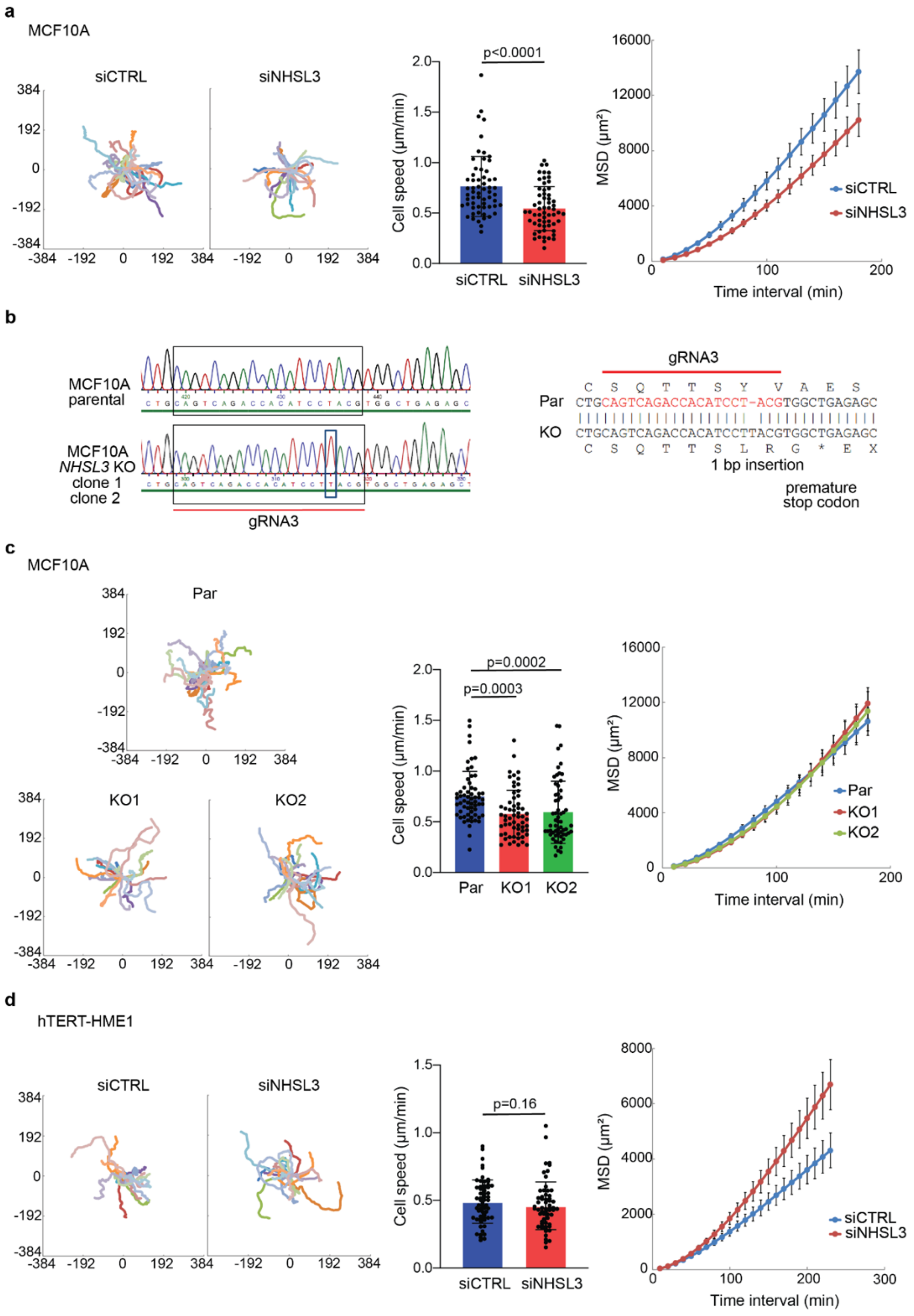

Supplementary Figure 1. a, related to Fig. 1a. Additional migration parameters of MCF10A cells upon NHSL3 knock-down. Trajectories, cell speed and Mean Square Displacement (MSD) of single cells. **b,** Genetic characterisation of the NHSL3 KO clones derived from MCF10A cells. Both clones contain the same 1 bp insertion that induces a frameshift in their two *NHSL3* alleles. **c, related to Fig. 1b.** Additional migration parameters of MCF10A cells upon NHSL3 KO. **d, related to Fig. 1c.** Additional migration parameters of hTERT-HME1 cells upon NHSL3 knock-down. Trajectory plots are labelled in µm. Data are presented as mean ± SEM. Statistical significance is calculated with Mann-Whitney test (**a, d**) or Kruskal-Wallis test (**c**) and p-values are indicated. Source data are provided as a Source Data file.

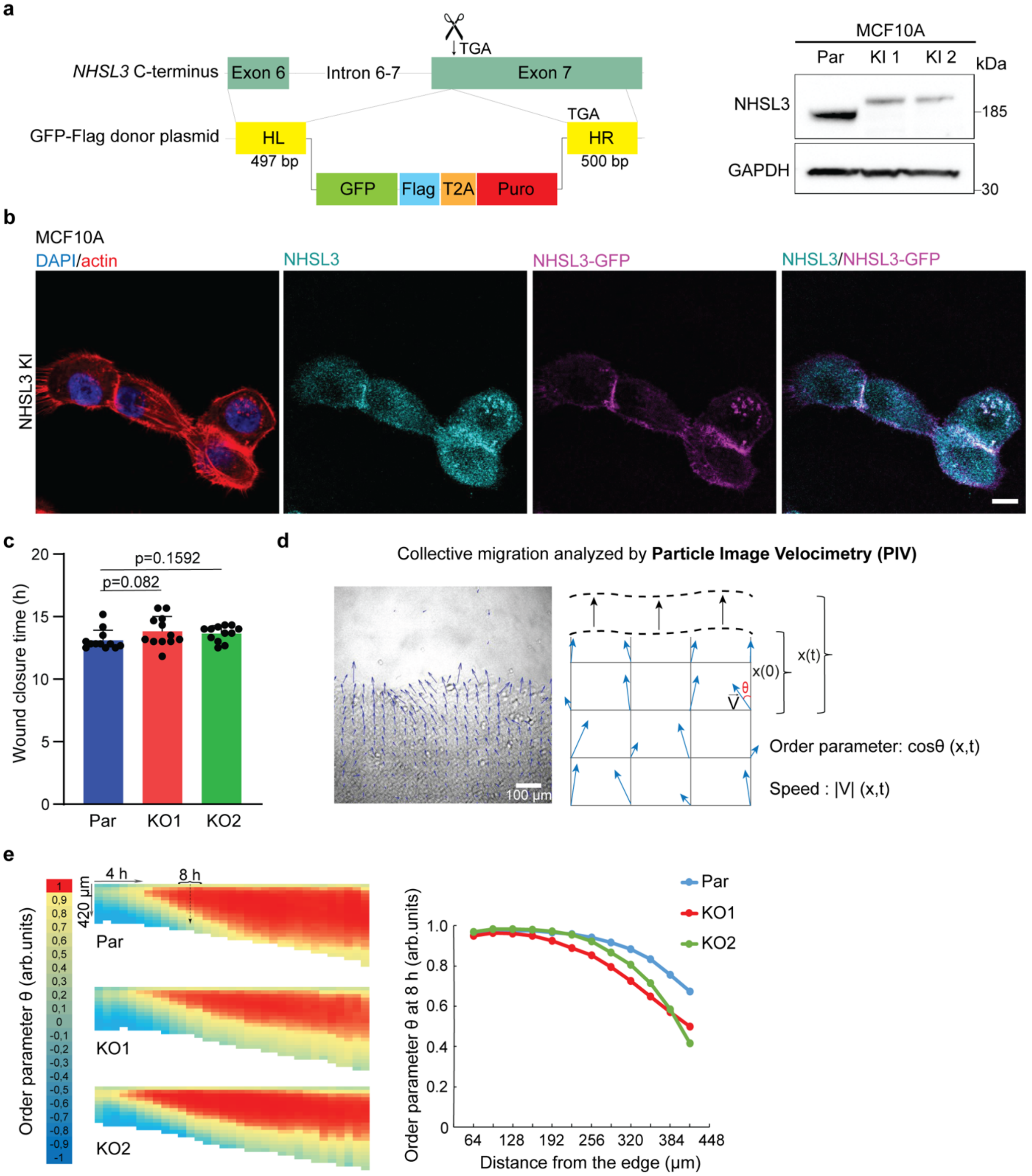

Supplementary Figure 2. a, related to Fig. 2e. Knock-in strategy used to introduce the dual GFP-Flag tag at the C-terminus of NHSL3 in MCF10A cells. The self-cleaving viral peptide T2A detaches the protein conferring puromycin resistance from the NHSL3-GFP-Flag fusion protein. The shifted NHSL3-GFP-Flag is detected in lysates from KI clones using NHSL3 antibodies in Western blot. **b,** KI cells expressing GFP-tagged endogenous NHSL3 are stained with DAPI, phalloidin and NHSL3 antibodies. The GFP signal overlaps with NHSL3 and stains cell-cell junctions. Single confocal section, scale bar: 10 µm. **c, relative to Fig. 1f.** Wound closure time of MCF10A parental (Par) and MCF10A NHSL3 KO clones (KO1, KO2). Data are presented as mean ± SEM. Statistical significance is calculated with Kruskal-Wallis test and

p-values are indicated. **d, relative to Fig. 1f.** Particle Image Velocimetry (PIV) provides a field of displacement vectors, whose amplitude provides speed and orientation provides the order parameter. **e, relative to Fig. 2i.** Heat maps of the order parameter of collective parental MCF10A or NHSL3 KO cells and order parameter at 8 h. Three biological repeats of all displayed experiments gave similar results. Source data are provided as a Source Data file.

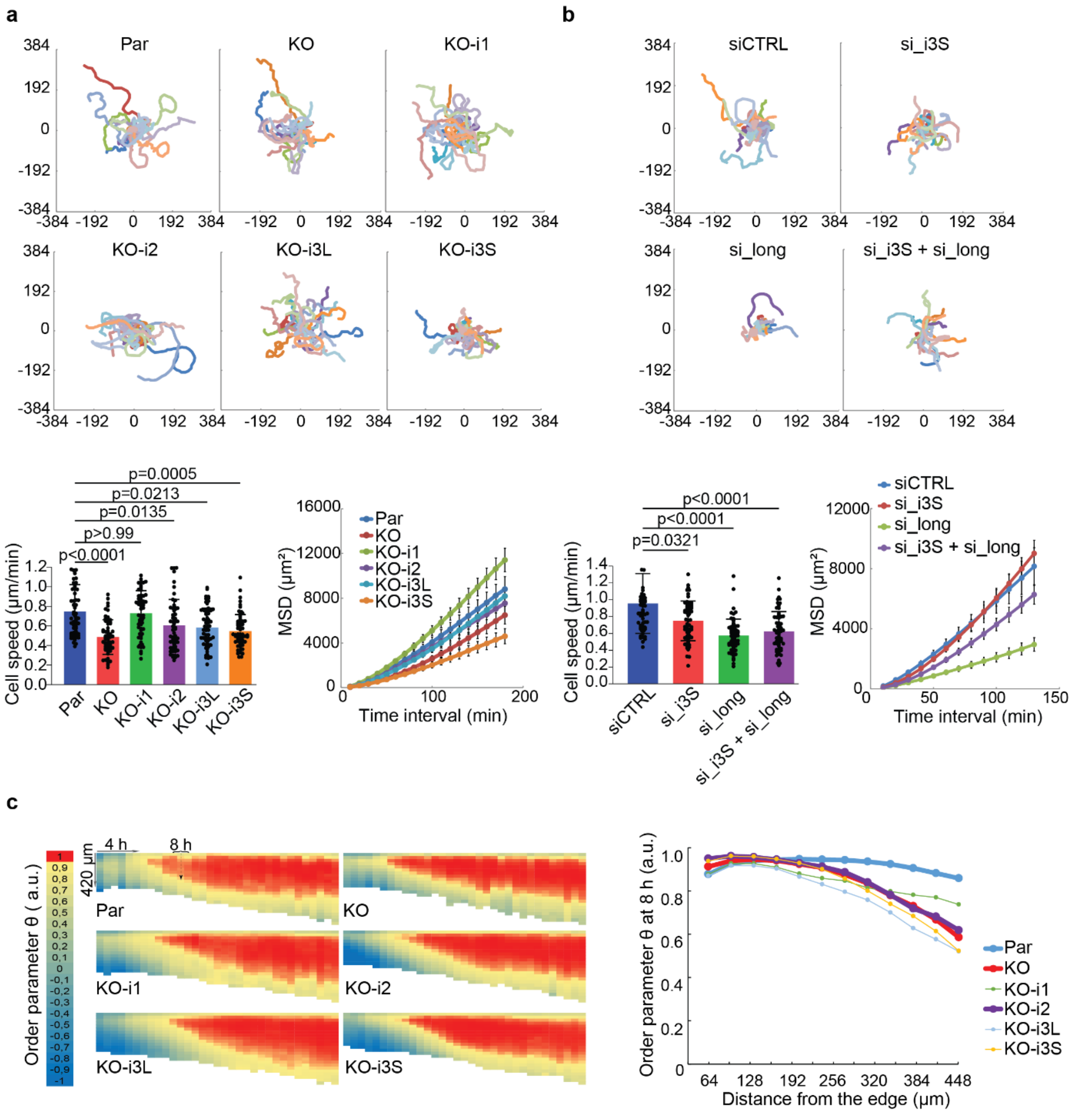

Supplementary Figure 3. a, related to Fig. 2c. Additional migration parameters of MCF10A cells upon NHSL3 KO and rescue. **b, related to Fig. 2h.** Additional migration parameters of MCF10A cells upon depletion of long and short isoforms of NHSL3. Trajectories, cell speed and Mean Square Displacement (MSD) of single cells. Data are presented as mean ± SEM. **c, relative to Fig. 2i.** Heat maps of the order parameter of collective parental MCF10A, NHSL3 KO or KO cells stably expressing NHSL3 isoforms and order parameter at 8 h. Trajectory plots are labelled in µm. Statistical significance is calculated with Kruskal-Wallis test and p-values are indicated. Source data are provided as a Source Data file.

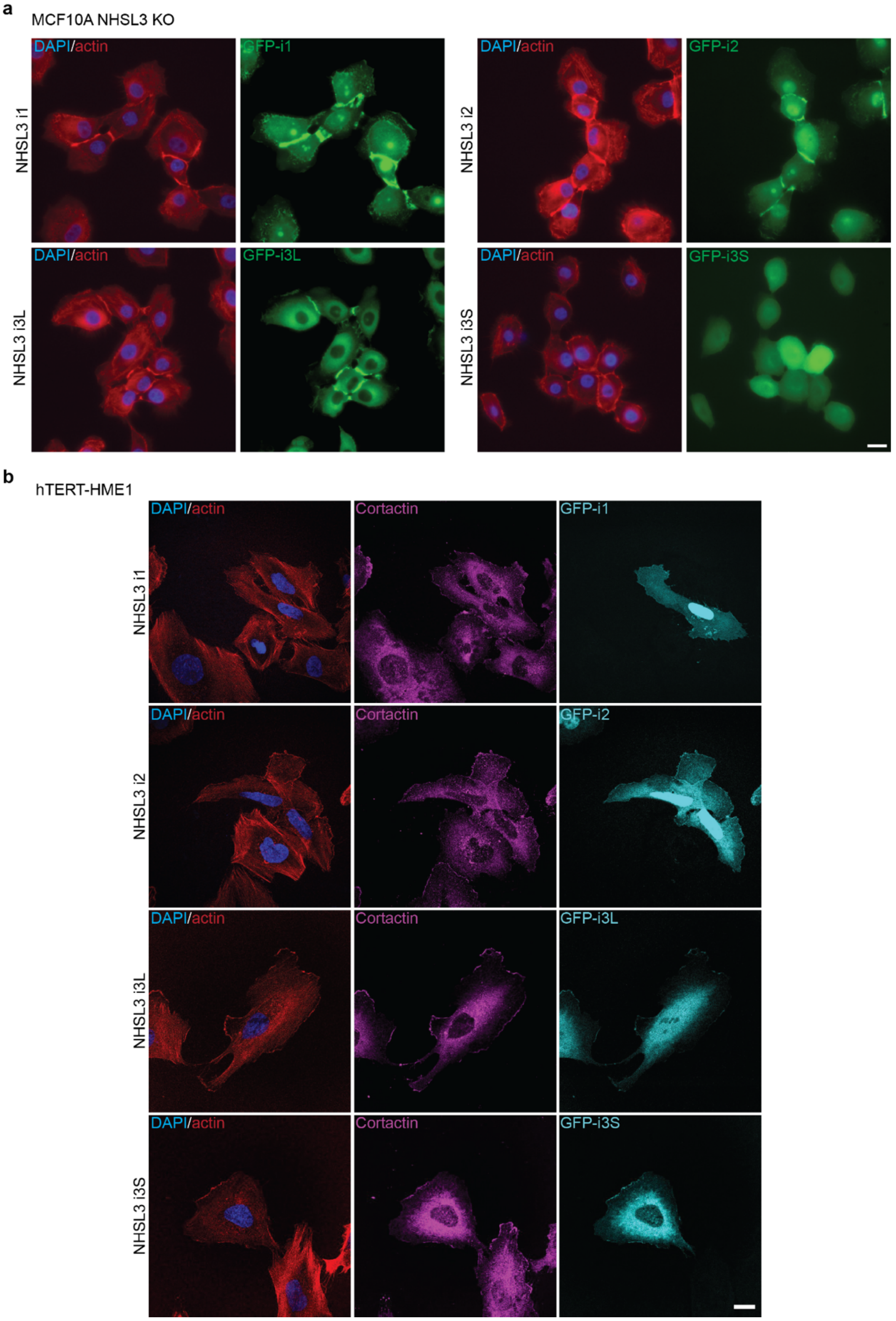

Supplementary Figure 4. Localisation of NHSL3 isoforms. a, MCF10A NHSL3 KO cells stably expressing the different Flag-GFP tagged isoforms are stained with DAPI and phalloidin. Scale bar: 20 µm. **b,** hTERT-HME1 cells are transiently transfected with the different Flag-GFP tagged isoforms are stained with DAPI, phalloidin and cortactin antibodies. The fluorescence of GFP is visualised. Single confocal section, scale bar: 20 µm.

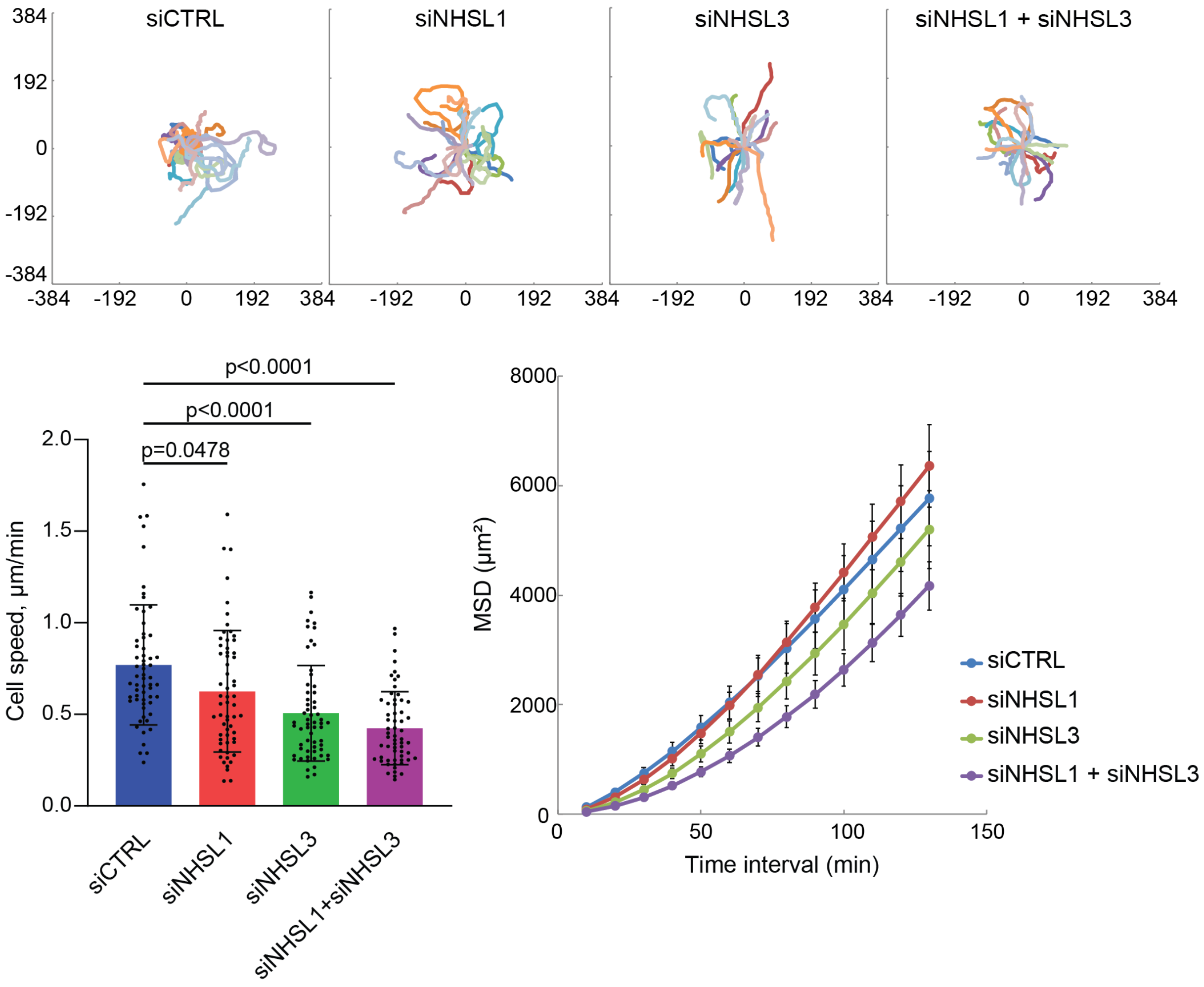

Supplementary Figure 5, related to Fig. 3a. Additional migration parameters of MCF10A cells transfected with siRNA targeting NHSL1 or NHSL3, alone or in combination. Trajectories, cell speed and Mean Square Displacement (MSD) of single cells. Trajectory plots are labelled in μm. Data are presented as mean ± SEM. Statistical significance is calculated with Kruskal-Wallis test and p-values are indicated. Source data are provided as a Source Data file.

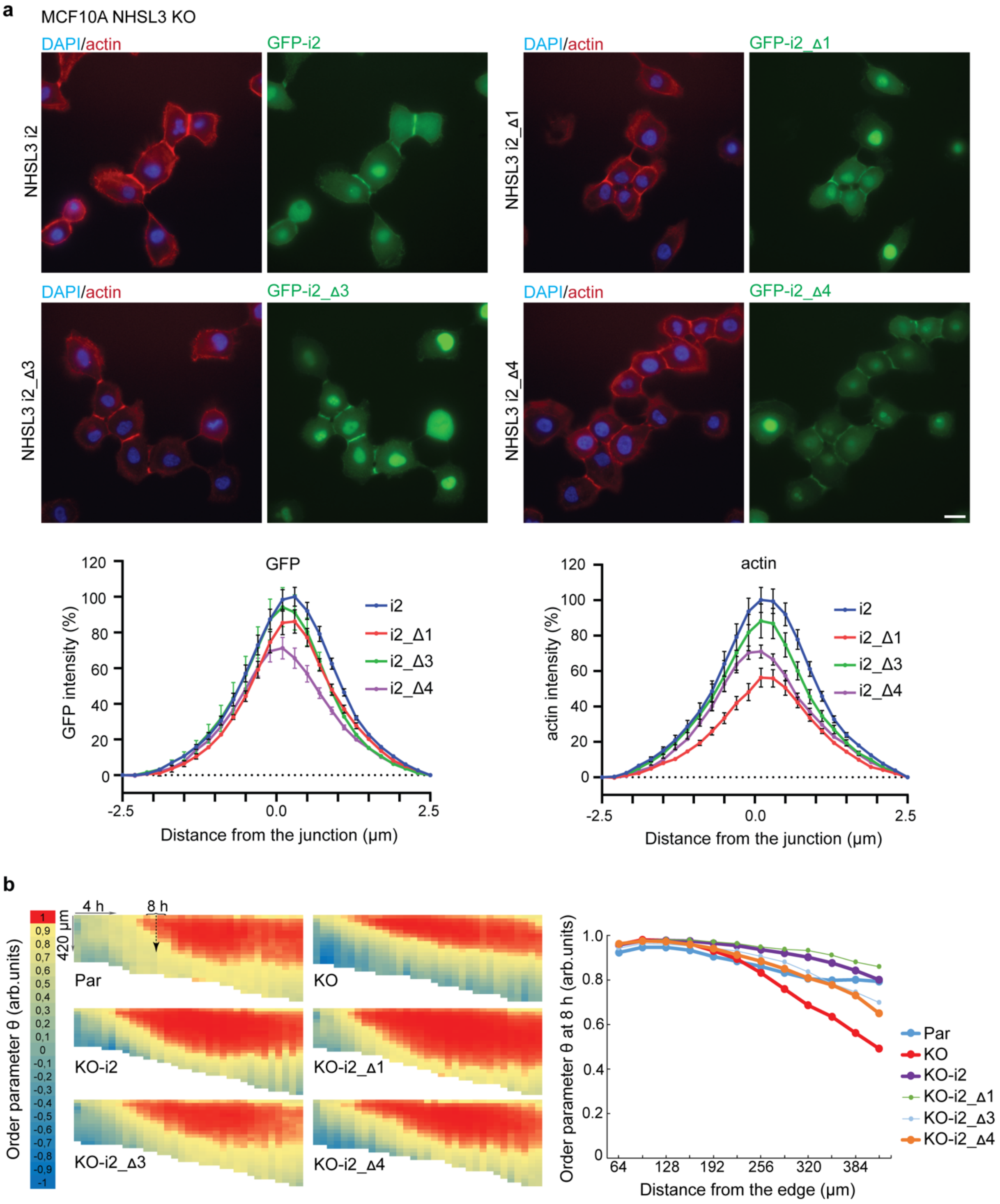

Supplementary Figure 6. a, MCF10A NHSL3 KO cells expressing different Flag-GFP tagged i2 isoforms deleted of partner binding sites stained with DAPI and phalloidin (actin). Scale bar: 20 µm. n=15, data are presented as mean ± SEM. **b, related to Fig. 5d.** Heat maps of the order parameter of collective parental MCF10A, NHSL3 KO or KO cells stably expressing the i2 isoform deleted of partner binding sites and order parameter at 8 h. Three biological repeats of each experiment gave similar results. Source data are provided as a Source Data file.

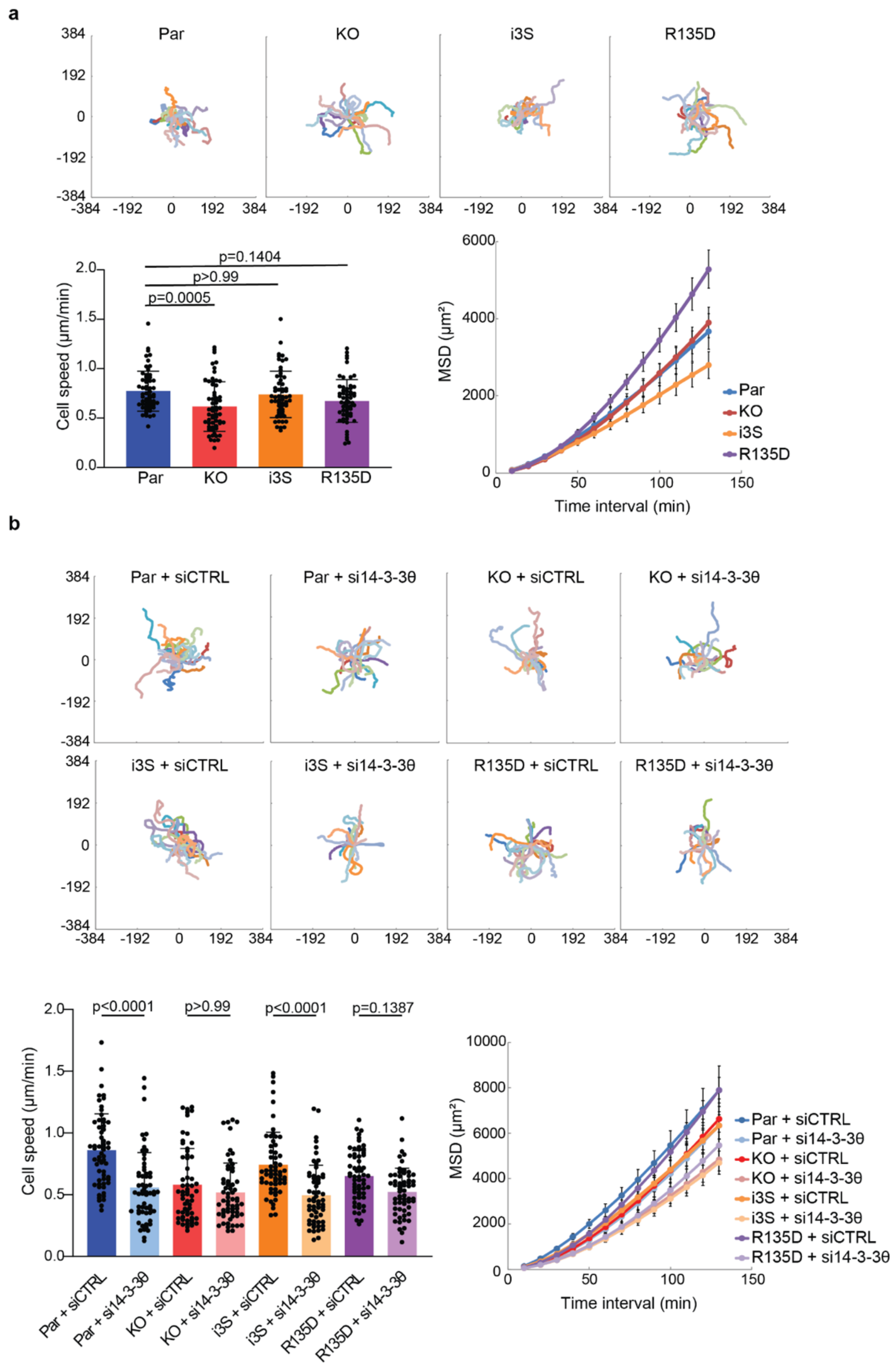

Supplementary Figure 7. a, related to Fig. 6f. Additional migration parameters of parental MCF10A and NHSL3 KO cells depleted or not of 14-3-3θ. **b, related to Fig. 6g.** Additional migration parameters of parental MCF10A, NHSL3 KO and KO cells stably expressing i3S or the R135D derivative are transfected with control or 14-3-3θ targeting siRNAs depleted or not of 14-3-3θ. Trajectories, Mean Square Displacement (MSD) and cell speed of single cells. Trajectory plots are labelled in µm. Data are presented as mean ± SEM. Statistical significance is calculated with Kruskal-Wallis test and p-values are indicated. Source data are provided as a Source Data file.

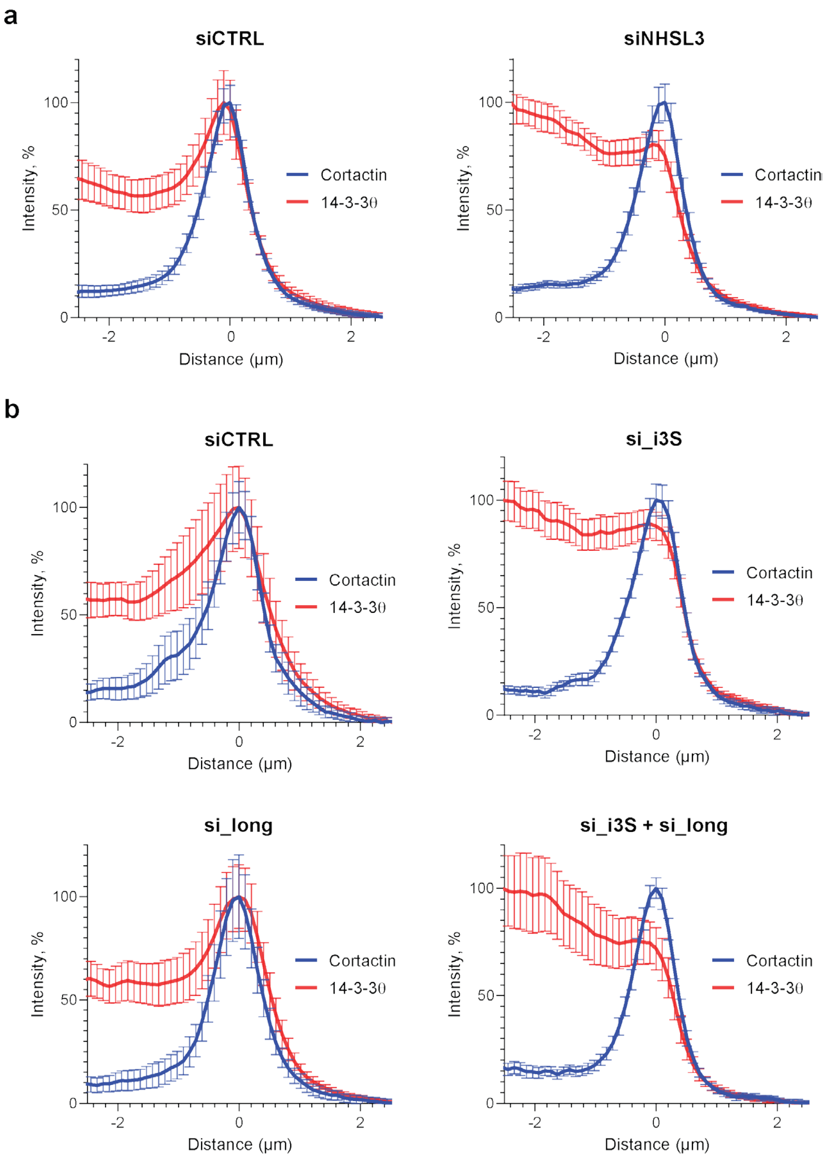

Supplementary Figure 8. a, related to Fig. 7a. Enrichment of 14-3-3θ in lamellipodia stained with cortactin in hTERT-HME1 cells treated with siCTRL or with siNHSL3. **b, related to Fig. 7b.** Enrichment of 14-3-3θ in lamellipodia stained with cortactin in hTERT-HME1 cells treated with siCTRL, si_i3S, si_long or with the combination of si_i3S + si_long. Multiple line scans (n = 15) registered to the cell edge. Data are shown as mean ± SEM. Three biological repeats of both experiments gave similar results. Source data are provided as a Source Data file.

Annex : Uncropped Western blots

Figure 1a

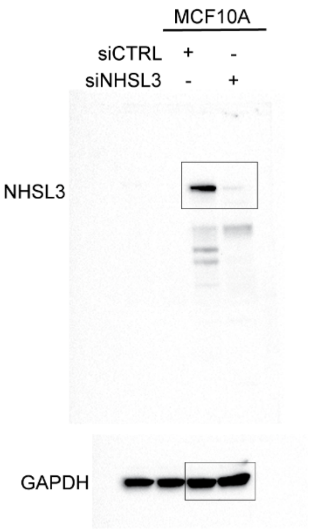

Figure 1b

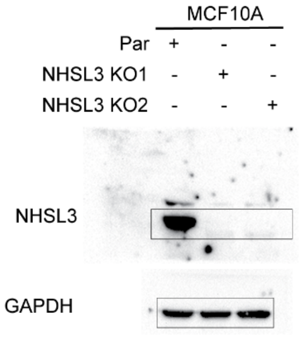

Figure 1c

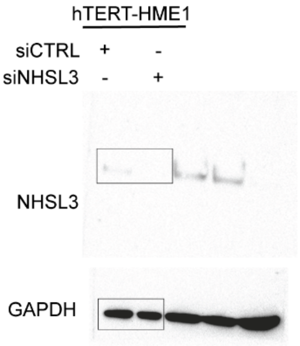

Supp Figure 2a

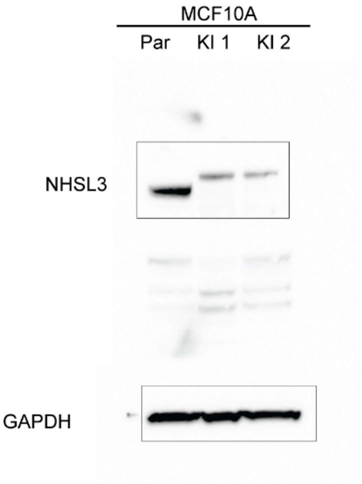

Figure 2b

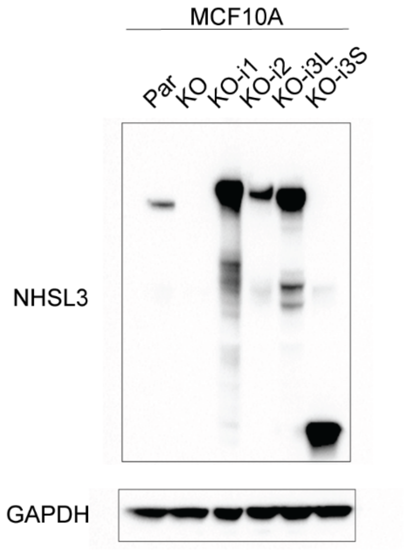

Figure 2e

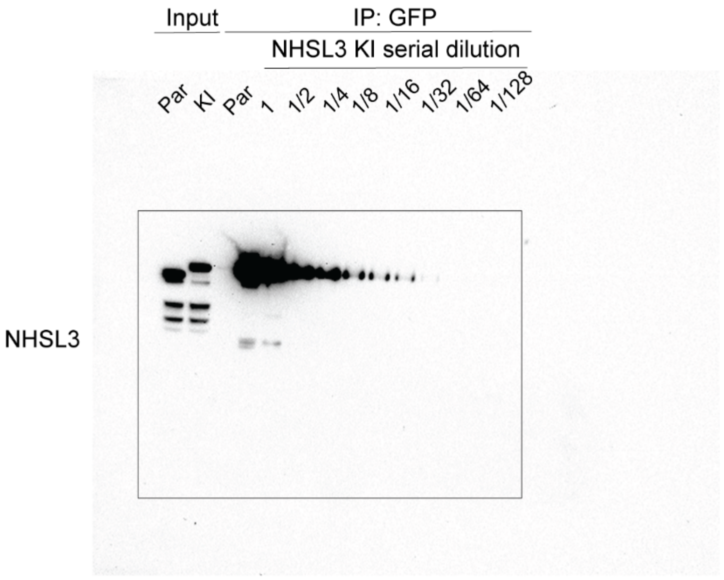

Figure 2f

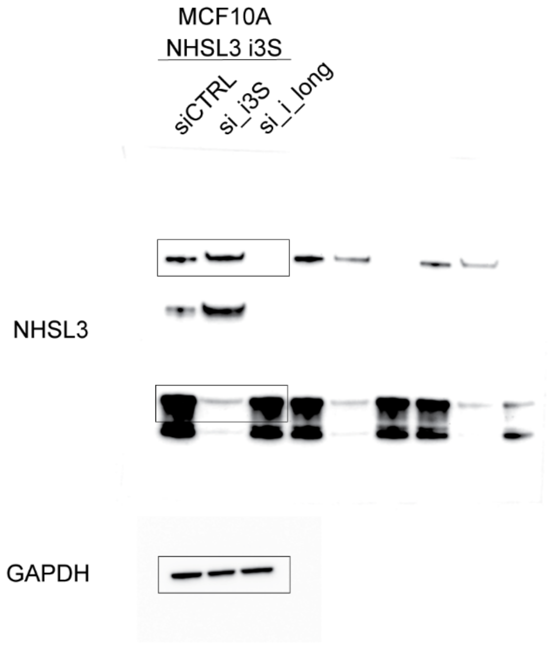

Figure 3a

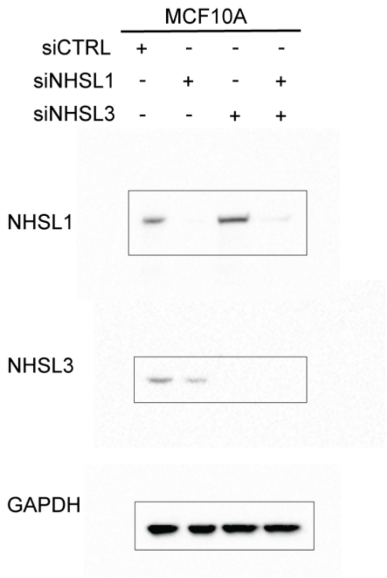

Figure 3b

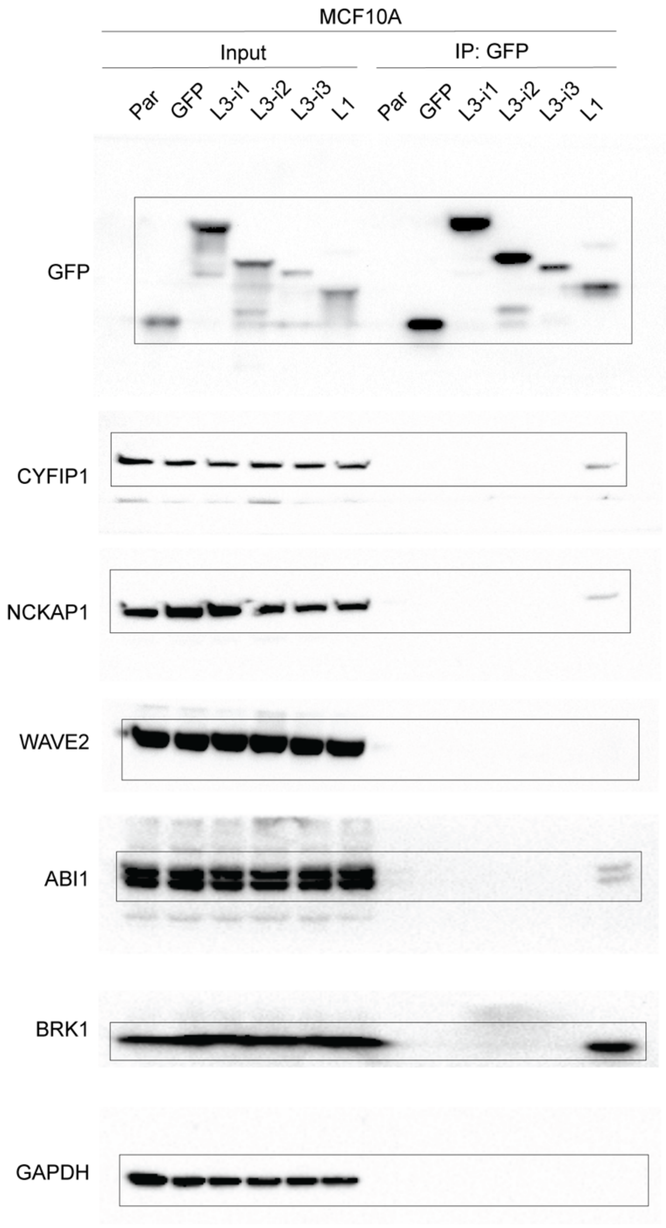

Figure 4a

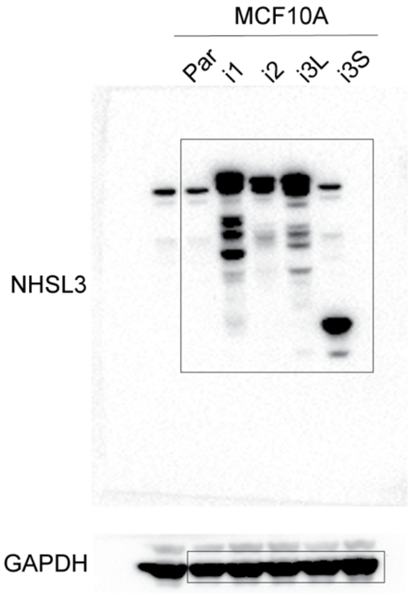

Figure 4d

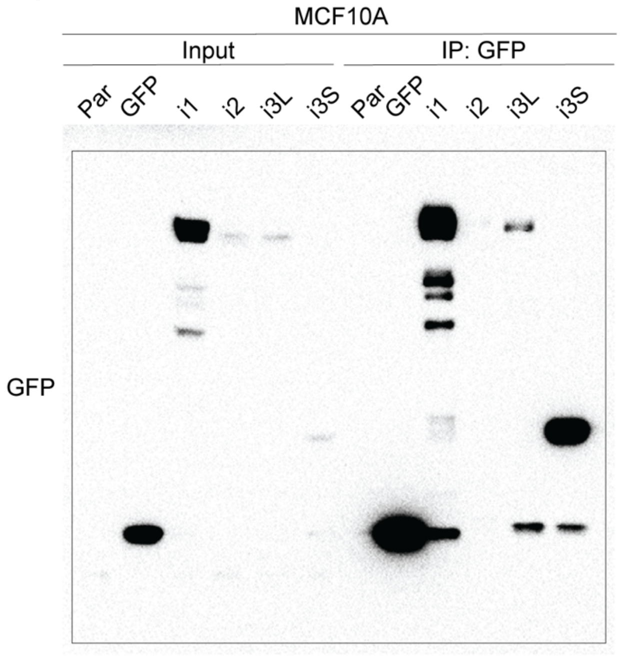

Figure 4d (continued)

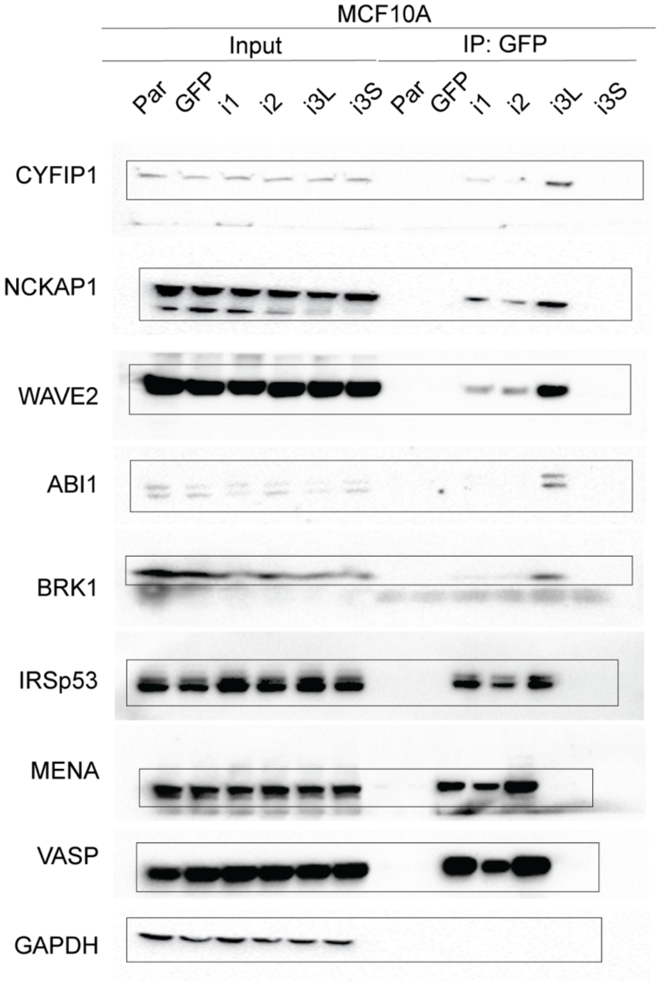

Figure 5c

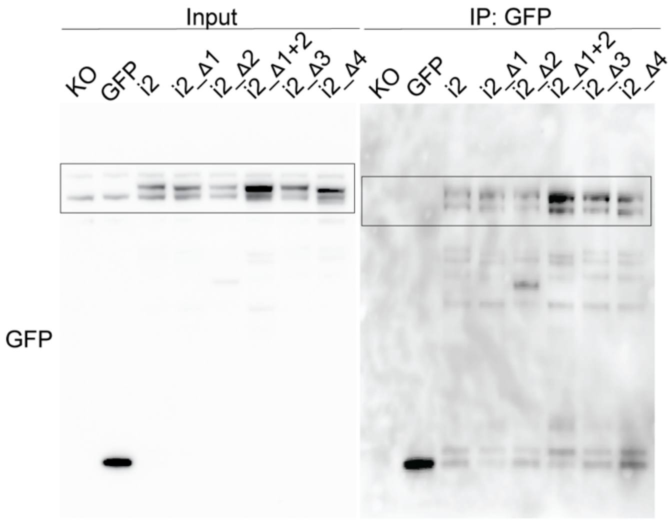
Input
IP: GFP
KO
GFP
i2
i2_Δ1
i2_Δ2
i2_Δ1+2
i2_Δ3
i2_Δ4
GFP

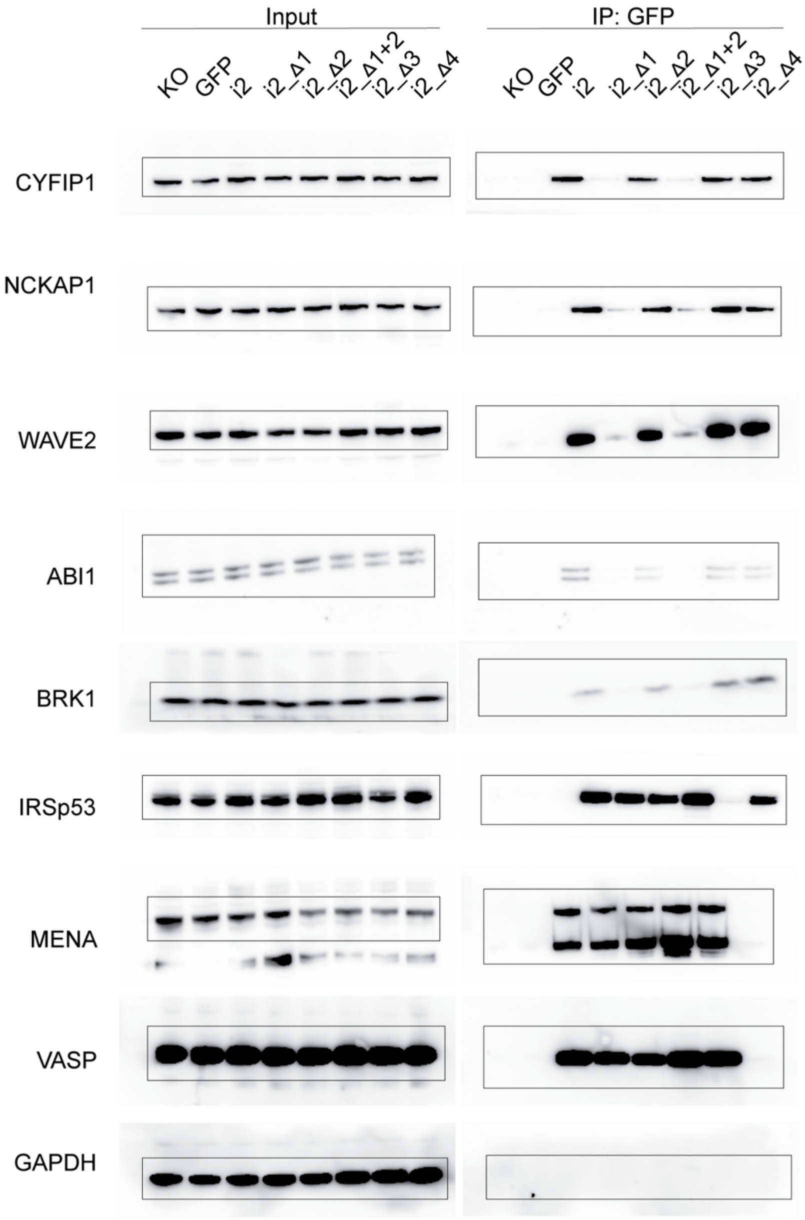
Input
IP: GFP
KO
GFP
i2
i2_Δ1
i2_Δ2
i2_Δ1+2
i2_Δ3
i2_Δ4
CYFIP1
NCKAP1
WAVE2
ABI1
BRK1
IRSp53
MENA
VASP
GAPDH

Figure 6b

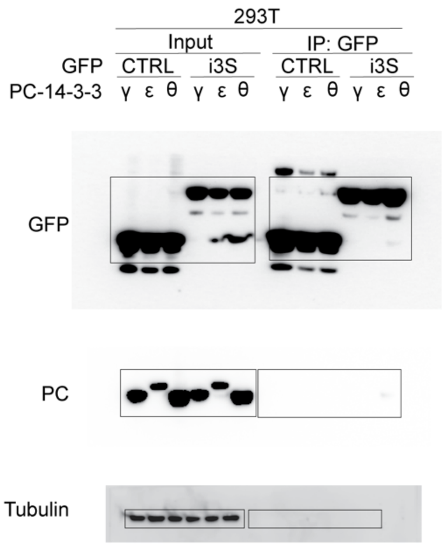

Figure 6d

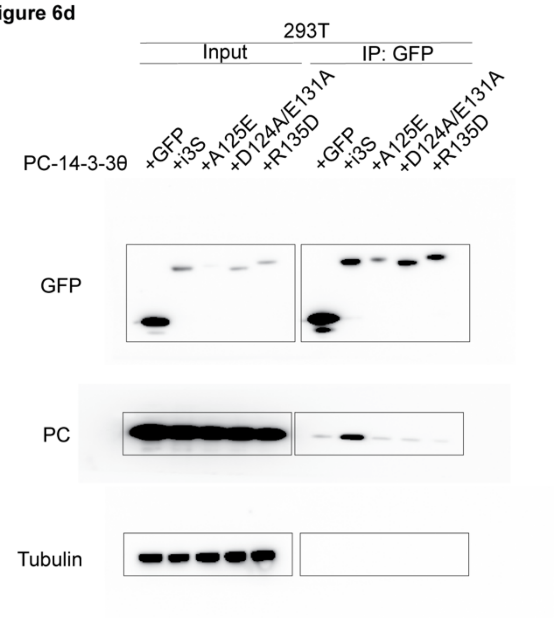

Figure 6e

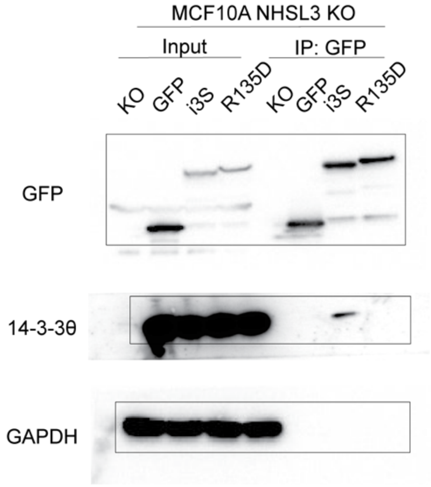

Figure 6g

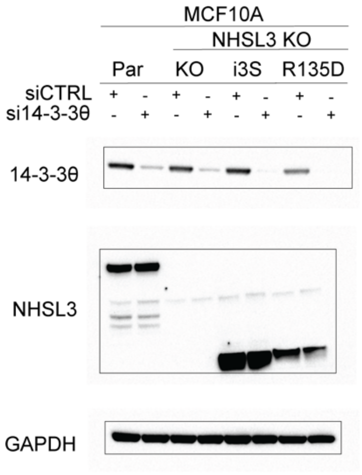

Figure 7a

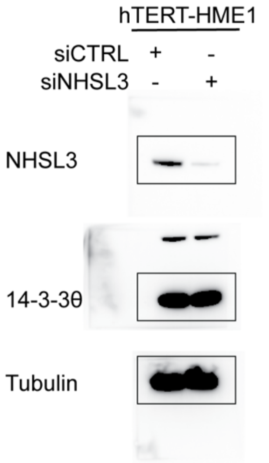

Supplement: Supplementary file 1 — Supplementary Information [file 41467_2024_55647_MOESM1_ESM.pdf]
