# Supplementary material for: NHSL3 controls single and collective cell migration through two distinct mechanisms
Source: Nat Commun. 2025 Jan 2;16:205. doi: 10.1038/s41467-024-55647-3 (PMC11696792; doi:10.1038/s41467-024-55647-3)
Supplement: Supplementary file 2 — Description of Additional Supplementary Information [file 41467_2024_55647_MOESM2_ESM.docx]

**Description of Additional Supplementary Files**

File Name: Supplementary Data 1

Description: All statistical results.

File Name: Supplementary Data 2

Description: Mass spectrometry data.

File Name: Supplementary Data 3

Description: Screening of structural models of putative interactions.

File Name: Supplementary Data 4

Description: Primers used in the study.

File Name: Supplementary Movie 1

Description: Related to figure 1a. Effect of NHSL3 depletion on migration persistence of MCF10A cells. MCF10A cells are transfected with control or NHSL3 siRNAs, recorded and tracked. Elapsed time in h and min. Scale bar: 20 μm

File Name: Supplementary Movie 2

Description: Related to figure 1b. Effect of NHSL3 knock-out on the migration persistence of MCF10A cells. Parental MCF10A and MCF10A NHSL3 KO cells are recorded and tracked. Elapsed time in h and min. Scale bar: 20 μm.

File Name: Supplementary Movie 3

Description: Related to figure 1c. Effect of NHSL3 depletion on migration persistence of hTERT-HME1 cells. hTERT-HME1 cells are transfected with control or NHSL3 siRNAs, recorded and tracked. Elapsed time in h and min. Scale bar: 20 μm.

File Name: Supplementary Movie 4

Description: Related to figure 1f. Effect of NHSL3 knock-out on wound healing of MCF10A cells. Parental MCF10A and MCF10A NHSL3 KO cells are recorded. Elapsed time in h and min after lifting the inset separating the two monolayers. Scale bar: 100 μm.

File Name: Supplementary Movie 5

Description: Related to figure 1f. Effect of NHSL3 knock-out on the displacement of follower cells in collective migration. Parental MCF10A and MCF10A NHSL3 KO cells are recorded, and subjected to PIV analysis. Elapsed time in h and min after lifting the inset separating the two monolayers. Scale bar: 100 μm

File Name: Supplementary Movie 6

Description: Related to figure 2c. Rescue of NHSL3 knock-out phenotype with isoform i3S. Parental MCF10A, NHSL3 KO cells and KO cells stably expressing i1, i2, i3L and i3S isoforms of NHSL3 are recorded and tracked. Elapsed time in h and min. Scale bar: 40 μm.

File Name: Supplementary Movie 7

Description: Related to figure 2h. Effect of depletion of short and long isoforms of NHSL3 on migration persistence of MCF10A cells. MCF10A cells are transfected with siRNAs targeting long and short isoforms, alone or in combination, recorded and tracked. Elapsed time in h and min. Scale bar: 40 μm

File Name: Supplementary Movie 8

Description: Related to figure 3a. Effect of combined depletion of NHSL1 and NHSL3 on migration persistence of MCF10A cells. MCF10A cells are transfected with siRNAs targeting NHSL1 and NHSL3, alone or in combination, recorded and tracked. Elapsed time in h and min. Scale bar: 40 μm.

File Name: Supplementary Movie 9

Description: Related to figure 6f. Effect of NHSL3 i3S and i3S_R135D on migration persistence of MCF10A NHSL3 KO cells. Parental MCF10A, NHSL3 KO cells and KO cells stably expressing wild type i3S or R135D i3S are recorded and tracked. Elapsed time in h and min. Scale bar: 40 μm.
