# Supplementary material for: NHSL3 controls single and collective cell migration through two distinct mechanisms
Source: Nat Commun. 2025 Jan 2;16:205. doi: 10.1038/s41467-024-55647-3 (PMC11696792; doi:10.1038/s41467-024-55647-3)
Supplement: Supplementary file 16 — Reporting Summary [file 41467_2024_55647_MOESM16_ESM.pdf]

nature portfolio

Corresponding author(s): Alexis Gautreau

Last updated by author(s): Jul 31, 2024

Reporting Summary

Nature Portfolio wishes to improve the reproducibility of the work that we publish. This form provides structure for consistency and transparency in reporting. For further information on Nature Portfolio policies, see our Editorial Policies and the Editorial Policy Checklist.

Statistics

For all statistical analyses, confirm that the following items are present in the figure legend, table legend, main text, or Methods section.

n/a	Confirmed	
[ ]	[x]	The exact sample size (*n*) for each experimental group/condition, given as a discrete number and unit of measurement
[ ]	[x]	A statement on whether measurements were taken from distinct samples or whether the same sample was measured repeatedly
[ ]	[x]	The statistical test(s) used AND whether they are one- or two-sided<br>*Only common tests should be described solely by name; describe more complex techniques in the Methods section.*
[x]	[ ]	A description of all covariates tested
[ ]	[x]	A description of any assumptions or corrections, such as tests of normality and adjustment for multiple comparisons
[ ]	[x]	A full description of the statistical parameters including central tendency (e.g. means) or other basic estimates (e.g. regression coefficient) AND variation (e.g. standard deviation) or associated estimates of uncertainty (e.g. confidence intervals)
[ ]	[x]	For null hypothesis testing, the test statistic (e.g. *F*, *t*, *r*) with confidence intervals, effect sizes, degrees of freedom and *P* value noted<br>*Give P values as exact values whenever suitable.*
[x]	[ ]	For Bayesian analysis, information on the choice of priors and Markov chain Monte Carlo settings
[x]	[ ]	For hierarchical and complex designs, identification of the appropriate level for tests and full reporting of outcomes
[x]	[ ]	Estimates of effect sizes (e.g. Cohen's *d*, Pearson's *r*), indicating how they were calculated

Our web collection on statistics for biologists contains articles on many of the points above.

Software and code

Policy information about availability of computer code

Data collection	Not applicable
Data analysis	DiPer, ImageJ, Graphpad Prism, Microsoft Excel, MAFFT, AlphaFold2, PIVlab, Matlab

For manuscripts utilizing custom algorithms or software that are central to the research but not yet described in published literature, software must be made available to editors and reviewers. We strongly encourage code deposition in a community repository (e.g. GitHub). See the Nature Portfolio guidelines for submitting code & software for further information.

Data

Policy information about availability of data

All manuscripts must include a data availability statement. This statement should provide the following information, where applicable:

- Accession codes, unique identifiers, or web links for publicly available datasets
- A description of any restrictions on data availability
- For clinical datasets or third party data, please ensure that the statement adheres to our policy

Raw files of the LC-MSMS analyses and database searches have been deposited in PRIDE (https://www.ebi.ac.uk/pride/) with the accession number PXD053609 and token 437mD8ut0jyp. The structural models of i) NHSL3_i3S-14-3-3θ (10.5452/ma-pptik), ii) NHSL3_i2-Abi1_site1 (10.5452/ma-pzi05), iii) NHSL3_i2-Abi1_site2 (10.5452/ma-e0o0t), iv) NHSL3_i2-IRSp53 (10.5452/ma-3kkf3), v) NHSL3_i2-MENA (10.5452/ma-dfoes), vi) NHSL3_i2-VASP (10.5452/ma-xrf33) are available in

ModelArchive (modelarchive.org) with the accession codes ma-pptik (password: 52h7T8ovhH), ma-pzi05 (password: jB8cg24VpC), ma-e0o0t (password: JJEofcFVNS), ma-3kkf3 (password: rQQiaqqaiT), ma-dfoes (password: Qvjwh98iFD) and ma-xrf33 (password: NOU7yRvXfc), respectively.

Research involving human participants, their data, or biological material

Policy information about studies with human participants or human data. See also policy information about sex, gender (identity/presentation), and sexual orientation and race, ethnicity and racism.

Reporting on sex and gender	not applicable
Reporting on race, ethnicity, or other socially relevant groupings	not applicable
Population characteristics	not applicable
Recruitment	not applicable
Ethics oversight	not applicable

Note that full information on the approval of the study protocol must also be provided in the manuscript.

Field-specific reporting

Please select the one below that is the best fit for your research. If you are not sure, read the appropriate sections before making your selection.

[x] Life sciences [ ] Behavioural & social sciences [ ] Ecological, evolutionary & environmental sciences

For a reference copy of the document with all sections, see nature.com/documents/nr-reporting-summary-flat.pdf

Life sciences study design

All studies must disclose on these points even when the disclosure is negative.

Sample size	Sample size was not predetermined by statistical methods. Statistical significance was evaluated retrospectively.
Data exclusions	No data were excluded
Replication	Technical and biological repeats are explicitly stated in figure legends. All results displayed were confirmed by several repeats without exclusion of potential negative experiments.
Randomization	Not applicable here. Control and treated samples were not randomized.
Blinding	Control and experimental groups were not random blinded to the investigator during data colection and analysis. This binding was not possible as most of the work presented here originates from the work of a single PhD student, the first author of the study.

Reporting for specific materials, systems and methods

We require information from authors about some types of materials, experimental systems and methods used in many studies. Here, indicate whether each material, system or method listed is relevant to your study. If you are not sure if a list item applies to your research, read the appropriate section before selecting a response.

Materials & experimental systems

n/a	Involved in the study	
[ ]	[x]	Antibodies
[ ]	[x]	Eukaryotic cell lines
[x]	[ ]	Palaeontology and archaeology
[x]	[ ]	Animals and other organisms
[x]	[ ]	Clinical data
[x]	[ ]	Dual use research of concern
[x]	[ ]	Plants

Methods

n/a	Involved in the study	
[x]	[ ]	ChIP-seq
[x]	[ ]	Flow cytometry
[x]	[ ]	MRI-based neuroimaging

Antibodies

Antibodies used	anti-NHSL3 (Sigma-Aldrich, HPA064839, 1:1000); anti-NHSL1 (Sigma-Aldrich, HPA029967, 1:1000); anti-GFP (Roche, 11814460001, 1:1000); anti-NCKAP1 (Bethyl Laboratories, A305-178A, 1:2000); anti-MENA (Sigma-Aldrich, HPA028696, 1:1000); anti-VASP (Sigma-

	Aldrich, HPA005724, 1:1000); anti-IRSp53 (Proteintech, 11087-2-AP, 1:1000); anti-14-3-3θ (Bethyl Laboratories, A303-146A, 1:1000); anti-GAPDH (Thermo Fisher Scientific, AM4300, 1:4000), anti-α-tubulin (Sigma T9026, 1:2000) and anti-cortactin (Sigma 05-180-I-100UL, 1:200). Home-made CYFIP1, ABI1, WAVE2 antibodies and BRK1 antibody were described previously, as stated in the manuscript.
Validation	Validation of all commercial antibodies were specified in respective commercial data sheets. The bands observed by Western blots using NHSL3 antibody were validated using knock-out or knock-down cells.

Eukaryotic cell lines

Policy information about cell lines and Sex and Gender in Research

Cell line source(s)	hTERT-HME1 (ME16C) and HEK 293T (CRL-3216) cells were from ATCC. MCF10A cell line was from the collection of breast cell lines organized by Thierry Dubois (Institut Curie, Paris).
Authentication	None of the cell lines were authenticated.
Mycoplasma contamination	All cell lines were routinely tested for mycoplasma infection and found to be negative.
Commonly misidentified lines (See ICLAC register)	No commonly misidentified cell lines were used in this study

Plants

Seed stocks	not applicable
Novel plant genotypes	not applicable
Authentication	not applicable
